# Interruption of the Right Pulmonary Artery in a Neonate

**DOI:** 10.1155/2022/7666677

**Published:** 2022-07-16

**Authors:** Mohammadreza Khalilian, Taraneh Faghihi Langroudi, Ali Dabbagh, Ramin Baghaei Tehrani, Tahmineh Tahouri

**Affiliations:** ^1^Department of Pediatrics, School of Medicine, Shahid Beheshti University of Medical Sciences, Tehran, Iran; ^2^Radiology Department, Shahid Modarres Hospital, Shahid Beheshti University of Medical Sciences, Tehran, Iran; ^3^Cardiac Anesthesia, Anesthesiology Research Center, Shahid Beheshti University of Medical Sciences, Tehran, Iran; ^4^Cardiac Surgery, Shahid Modarres Educational Hospital, School of Medicine, Shahid Beheshti University of Medical Sciences, Tehran, Iran

## Abstract

Interruption of the right pulmonary artery is a very rare anomaly which can be associated with other congenital heart lesions or can occur in isolation. Clinical presentations of the unilateral interruption of a pulmonary artery are varied including pulmonary hypertension, recurrent infection, dyspnea, exercise intolerance, hemoptysis, and chest pain. Less commonly, patients may be asymptomatic. Diagnosis of this anomaly is made by echocardiography and CT angiography as well as cardiac MRI. Treatment options are medical versus surgical management and often recommended in symptomatic patients with pulmonary hypertension, recurrent infection, and hemoptysis. Herein, we describe a very rare case of right pulmonary artery originating from the right subclavian artery in a 12-day-old neonate.

## 1. Introduction

Normally, the pulmonary artery originates from the right ventricle and bifurcates into the right and left pulmonary artery branches (1). Anomalous origin of the right and left pulmonary arteries is a rare congenital anomaly which is usually accompanied by other congenital heart diseases; however, it can occur in isolation. Interruption of the right pulmonary artery is one of the extremely rare branch pulmonary artery anomalies which occur due to abnormal development of the proximal part of the sixth primitive aortic arch. In this condition, perfusion of the right lung is through collateral arteries arising from the systemic circulation (2). Needless to say, the right and left pulmonary arteries are nonconfluent, and the left pulmonary artery is normally connected to the right ventricle and thus receives the entire cardiac output (3, 4).

Herein, we describe a very rare case of an isolated right pulmonary artery originating from the right subclavian artery in a 12-day-old neonate.

## 2. Case Presentation

A 12-day-old newborn girl was referred to our hospital, Shahid Modarres Educational Hospital, located in Tehran, Iran, from a district hospital for further evaluation of pulmonary hypertension. She was delivered by cesarean section at term gestation in a twin pregnancy and had APGAR scores of 9/10 and a birth weight of 2100 g. Shortly after birth, she developed tachypnea and was consequently transferred to the neonatal intensive care unit. The second twin was healthy. The parents were Pakistani. The mother of the patient did not take any medications during pregnancy. The pregnancy was uneventful, and there was no relevant familial history of consanguinity or congenital heart defects. The patient was diagnosed with pulmonary hypertension, and medical treatment was initiated with sildenafil. She was discharged after 11 days in stable condition.

After discharge, the patient was referred to our Pediatric Cardiology Clinic in a tertiary center. Physical examination on admission revealed a respiratory rate of 45 cycle/minute, BP of 85/50 mmHg, and heart rate of 120 beat/minute. She had no respiratory distress, and oxygen saturation was 94% in room air. Cardiac auscultation revealed a systolic murmur on the left upper sternal border. Examination of other body system was normal. Electrocardiogram (ECG) showed normal sinus rhythm and right axis deviation. Chest X-ray indicated mild cardiomegaly with mild increase in pulmonary vascular markings suggestive of pulmonary hypertension.

**Figure 1:**
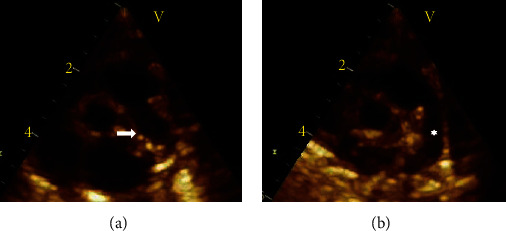
Cross-sectional echocardiogram (parasternal short axis view) showing (a) the LPA at its typical location (arrow) and absence of the RPA and (b) large left side PDA (asterisk). LPA: left pulmonary artery. RPA: right pulmonary artery.

**Figure 2:**
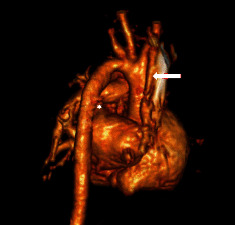
3D reconstruction CT imaging from right posterior view shows the origin of the right pulmonary artery from the right subclavian artery (white arrows) and the left-sided PDA (asterisk). The stenosis of the RPA is also seen.

**Figure 3:**
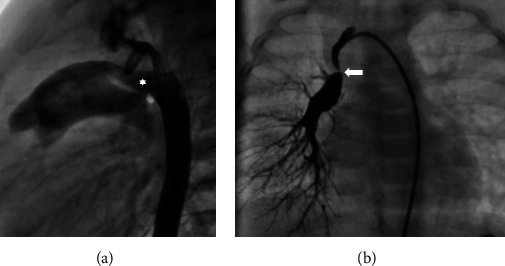
Angiogram showing (a) a large size patent ductus arteriosus (asterisk), and (b) the right pulmonary artery originates from the right subclavian artery with a significant stenosis at the origin point (arrow).

An echocardiogram was notable for mild right atrial and right ventricular enlargement. The main pulmonary artery was mildly dilated, and the typical bifurcation of the pulmonary artery was not detectable; instead, only the left pulmonary artery was seen at its expected location, and the right pulmonary artery was absent. The pulmonary valve was normal. A large PDA with bidirectional shunt was noted with a typical insertion to the LPA (Figure 1). There was a mild tricuspid regurgitation and estimated a systolic pulmonary artery pressure of 50 mmHg above right atrial pressure. There was mild right ventricular diastolic dysfunction on M-mode and tissue Doppler evaluation. Otherwise, echocardiogram was normal with no other cardiac anomaly except for a small patent foramen ovale which had a bidirectional shunt too. Since we could not see the right pulmonary artery on the echocardiogram, cardiac CT angiography was performed which demonstrated an isolated right pulmonary artery arising from the right subclavian artery (Figure 2). Since the patient was mildly symptomatic, she was discharged from the hospital with no medications and a plan for monthly follow-up. At the age of 3 months, the patient underwent angiography and catheterization. At cardiac catheterization, the left pulmonary artery pressure was 80/50 mmHg versus an aortic pressure of 90/50 mmHg. There was a significant step up from the right ventricle at 82% to the left pulmonary artery at 99%. Aortogram subsequently revealed a large left tubular patent ductus arteriosus. The origin of the right pulmonary artery was demonstrated from the right subclavian artery with a significant proximal stenosis at the origin from the right subclavian artery (Figure 3). The size of the right pulmonary artery was within normal ranges while the left pulmonary artery was significantly dilated.

**Figure 4:**
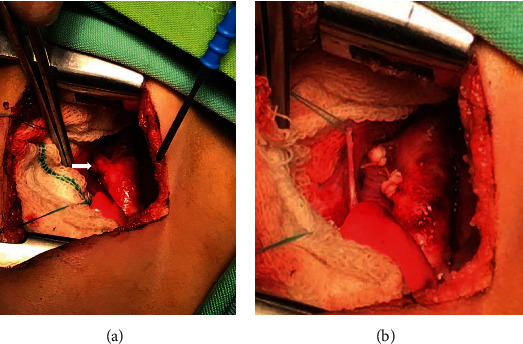
Intraoperative images: (a) large size patent ductus arteriosus was obvious (arrow); (b) ligation of the PDA was performed.

The patient subsequently underwent surgical PDA ligation (Figure 4). It was felt that the proximal RPA stenosis reduced the risk of arterial hypertension in the right lung, so no intervention was performed on the right pulmonary artery at that time. The postoperative course was uneventful, and she was discharged after 6 days with a recommendation for continued follow-up. She was also started on medical therapy with sildenafil and furosemide which in the months that followed was discontinued. At present, the patient is 8 months old and remains asymptomatic with an estimated pulmonary artery pressure of 20 mmHg on echocardiography and normal growth and development.

## 3. Discussion

Interruption of the right pulmonary artery is a very rare anomaly with a prevalence of 1 : 200,000 to 1 : 300,000 in adult, which was first described in 1868 by Frantzel O. Angeborener. This anomaly has been described in literature as unilateral absence of the pulmonary artery; however, the distal intrapulmonary network is usually intact in these cases and perfused by collateral vessels. Therefore, the term “interruption” rather than “absence” is the preferred term (5, 6). To our best knowledge, the case herein represents the fourth definite diagnosis of neonatal isolated interrupted right pulmonary artery reported in literature. The three other cases refers to a two-day-old female presenting with a murmur, a one-day-old neonate presenting with severe cyanosis, and a two-day-old neonate presenting with cyanosis and oxygen saturation of 80% (5, 7).

Interruption of the right pulmonary artery is commonly associated with other congenital heart defects such as truncus arteriosus and tetralogy of Fallot. In isolated forms which mostly concerns the right pulmonary artery, it often involves the pulmonary artery branch contralateral to the aortic arch (5, 8).

Review of the literature revealed that abnormal right pulmonary artery origin can be of three types. In the first type, the right or left pulmonary artery arises from the ascending aorta; in the second type, the proximal portion of the left or right pulmonary artery is absent, and the distal part is supplied by a patent ductus arteriosus, and the last type is absence of left or right pulmonary artery, and the supply of distal pulmonary artery branch is through aortopulmonary collaterals (9). Our patient had interruption of the right pulmonary artery, and the origin of the distal part was from the right subclavian artery which can be placed in the second category.

Clinical presentations of the unilateral interruption of a pulmonary artery can be varied. Pulmonary hypertension, dyspnea, recurrent pulmonary infections or chest pain, exercise intolerance, and hemoptysis in older patients have been reported. However, in rare condition, patients can be asymptomatic and remain undiagnosed until adulthood (10). At the time of our patient's presentation, she had signs and symptoms of pulmonary hypertension. Indeed, the patient's main pulmonary artery seemed mildly dilated in both echocardiography and CT angiography, and her oxygen saturation was decreased to 92%. The cause of pulmonary hypertension, which can be present in about 40-45% of unilateral absence of a pulmonary artery cases, is the fact that the entire cardiac output is directed to the contralateral lung leading to endothelial injury and vasoconstrictor mediator release (11).

Currently, there is no consensus as to the treatment of unilateral interruption of a pulmonary artery. Treatment is often recommended in symptomatic patients with pulmonary hypertension, recurrent infection, or hemoptysis. Medical treatment with pulmonary vasodilators or surgical management, including total or staged pulmonary artery anastomosis partial or complete pneumonectomy, has been suggested. Basically, in the presence of an isolated pulmonary artery branch, if the size of intrapulmonary branches is within normal range, an anastomosis with the central pulmonary artery is the best surgical approach. In the patients with small intrapulmonary arteries, a modified Blalock-Taussig shunt is recommended before the anastomosis to aid with vessel growth prior to unifocalization. In asymptomatic patients who have no cardiopulmonary dysfunction, clinical follow-up may be reasonable (12, 13). Our patient had pulmonary hypertension; therefore, PDA ligation was performed in hopes of decreasing the pulmonary artery pressure. The right pulmonary artery was normal in size, and there was no significant reduction in perfusion of the right lung on the chest X-ray. On the other hand, there was a significant stenosis at the origin of the right pulmonary artery which limits the blood flow from the subclavian and therefore reduces the risk of pulmonary hypertension in the right lung. Accordingly, our pediatric cardiology team decided not to perform any intervention on the right pulmonary artery. Accordingly, the patient's follow-up at regular intervals showed that she was asymptomatic. However, since the connection between the right subclavian artery and the right pulmonary artery, which allows degrees of left to right shunt, remains, it seems necessary to measure left ventricular volume over times.

In conclusion, since the isolated interruption of the right pulmonary artery is a very rare congenital anomaly, it can be difficult to diagnose. Early diagnosis in neonatal period and treatment, if necessary, will hopefully prevent significant morbidity and mortality in these patients.

## Data Availability

Data sharing is not applicable to this article as no new data were created or analyzed in this study.

## References

[1] Carter B. W., Lichtenberger J. P., Wu C. C. (2014). Congenital abnormalities of the pulmonary arteries in adults. *American Journal of Roentgenology*.

[2] Bueno J., Flors L., Mejía M. (2017). Congenital anomalies of the pulmonary arteries: spectrum of findings on computed tomography. *Radiologia (English Edition)*.

[3] Agati S., Sousa C. G., Calvaruso F. D. (2019). Anomalous aortic origin of the pulmonary arteries: case series and literature review. *Pediatric Cardiology*.

[4] Garg P., Talwar S., Kothari S. S. (2012). The anomalous origin of the branch pulmonary artery from the ascending aorta. *Interactive cardiovascular and thoracic surgery*.

[5] Raymond A., Pedretti E., Privitera G., Cicero C., Biasucci G. (2018). Neonatal diagnosis of isolated absence of the right pulmonary artery: a case report and review of the literature. *Italian Journal of Pediatrics*.

[6] Anand S. H., Jasper A., Mani S. E., Joseph E., Mathai J. (2015). Proximal interruption of the pulmonary artery: a case series. *Journal of Clinical and Diagnostic Research: JCDR*.

[7] Kardos M., Kaldararova M., Ondriska M. (2014). Double ductus arteriosus and anomalous origin of the right pulmonary artery from the right-sided duct. *Journal of Cardiology Cases.*.

[8] Cortinas E. C., Jiménez N. R., Zurita M. B., Haro P. M., Silva L. G. (2019). Unusual distal right pulmonary artery origin from right ductus arteriosus with uncommon left-sided aortic arch. *Revista Colombiana de Cardiología*.

[9] Penkoske P. A., Castañeda A. R., Fyler D. C., Van Praagh R. (1983). Origin of pulmonary artery branch from ascending aorta. *The Journal of Thoracic and Cardiovascular Surgery*.

[10] Reading D. W., Oza U. (2012). Unilateral absence of a pulmonary artery: a rare disorder with variable presentation. *In Baylor University Medical Center Proceedings*.

[11] Saladi L., Roy S., Diaz-Fuentes G. (2018). Unilateral pulmonary artery agenesis: an unusual cause of unilateral ARDS. *Respiratory Medicine Case Reports*.

[12] Weldetsadik A. Y., Asfaw Y. M., Tekleab A. M. (2018). Isolated absence of right pulmonary artery in a 4-year old child: a case report. *International Medical Case Reports Journal*.

[13] Singhi A. K., Francis E., Kumar R. K. (2010). Isolated absence of right pulmonary artery. *Annals of Pediatric Cardiology*.

